# Cancer education matters: a report on testicular cancer knowledge, awareness, and self-examination practice among young Polish men

**DOI:** 10.1038/s41598-020-77734-3

**Published:** 2020-11-26

**Authors:** Łukasz Pietrzyk, Marta Denisow-Pietrzyk, Marcin Czeczelewski, Konrad Ślizień-Kuczapski, Kamil Torres

**Affiliations:** 1 Department of Didactics and Medical Simulation, Medical University of Lublin, Lublin, Poland; 2 Department of General, Oncological, and Minimally Invasive Surgery, 1st Military Clinical Hospital With the Outpatient Clinic in Lublin, Lublin, Poland; 3 Students’ Scientific Association of Medical Simulation, Department of Didactics and Medical Simulation, Medical University of Lublin, Lublin, Poland

**Keywords:** Health care, Diseases, Cancer

## Abstract

The objective of the study was to assess the level of testicular cancer (TC) knowledge and awareness as well as the altitude and practice towards testicular self-examination (TSE) among Polish male high school and medical students. An original questionnaire survey was conducted in SE Poland with a representative sample of 1077 male students: 335 from high school and 742 medical students. The results indicate the knowledge about TC epidemiology and the awareness of risk factors responsible for the TC was low. The knowledge of the signs and symptoms of TC was significantly higher among the medical students, compared to the high school students. The level of education was associated with the awareness of methods for early detection and symptoms of TC. A satisfactory level of awareness of the TSE practice was exhibited only by the medical students. The main reason for not performing self-examination was the lack of knowledge and practical skills. The deficits of knowledge of TC in young men should motivate the education policy makers in Poland to implement education in the field of TC issues more widely in high schools. Moreover, cancer prevention modules and/or teaching methods should be improved in medical schools.

## Introduction

Testicular cancer (TC) is the most common malignancy and the third leading cause of cancer deaths in men between 15–40 years^1,2^. It is a growing public health concern, as the incidence rates of TC have systematically increased worldwide in the last decades. Annually, 50,000 new cases and 10,000 deaths have been noticed globally^3^. Population-based epidemiological studies revealed ethnic differences in the TC epidemiology. The highest incidence rate was recorded among Caucasian individuals and in Nordic countries, where the incidence rates of TC were tenfold higher compared to the Asian and African populations^4,5^.

Nowadays, there is no primary method to prevent TC^5,6^. The main cause of TC deaths results from a delayed diagnosis and detection of the cancer at an advanced metastatic stage^4,6^. In the European population, the 5-year survival rate is higher than 90% for TC patients being diagnosed at an early stage^7^.

Early detection of testicular cancer has an important role in reducing the mortality, increasing the survival rates, and prognosis of the disease^7^. However, there is no expert consensus on screening for TC. For example, the National Cancer Institute and the U.S. Preventive Service Task Force do not recommend TC screening due to inadequate evidence confirming its detection power, while data from the American Cancer Society or Cancer Research UK endorse a screening method to support early TC detection (). Although the risk and benefits for testicular self-examination (TSE) are debatable, this method can potentially help to diagnose TC at the early stage^8–13^.

Performing regular TSE may also have other benefits, i.e. men can become familiar with their own anatomy, gain knowledge how to detect and monitor the abnormalities other than cancer, and consider quicker contact with health care personnel to consult the detected anomalies^14^. However, the knowledge and awareness of TC and TSE is very poor among young men worldwide^1–9,14–17^. Interestingly, the gap in the knowledge is reported even among men that have completed the higher level of education, including health care or medical education^18^.

TC awareness and knowledge seems to be linked to a smaller tumor size at presentation, higher curability, and reduced treatment costs. Therefore, the primary purpose of strategies to prevent TC should be implementation of educational programs designed to increase awareness and knowledge^7^. Moreover, the programs should encourage men to be aware of pathological processes in male genitalia in order to reduce cancer-related deaths^6,7^. To increase men’s response to programs enhancing men’s awareness of TC, all interventions should be brief, catchy, and visually stimulating with technology involvement. Most importantly, to strengthen the acceptance for screening, men should be involved in designing effective educational projects/initiatives, as the more active levels of co-designing, the more engagement in health promotion^19^.

Several national campaigns have already been undertaken to promote a healthier lifestyle and change on individual's behavior, i.e. ‘Movember’, which subsidized over 1200 projects in about 20 countries. The projects were aimed at early detection of cancer, promotion of self-examination practices, and implementation of effective treatments^20,21^.

The aim of the study was to assess the level of testicular cancer knowledge and awareness as well as the attitude and practice towards testicular self-examination among Polish male high school and medical students.

## Results

### Characteristics of the male participants

All students selected for the survey participated in the study, completed the questionnaire, and their answers were evaluated. The mean age of the participants was 19.4 years ± 2.09 SD; range: 14–26 years. The high school students accounted for 31.1% (n = 335), while the medical students represented 68.9% (n = 742). In the group of the medical students, 55.8% (n = 414) were attending their pre-clinical training and 44.2% (n = 328) were from the clinical training subgroup. The majority (87.2%; n = 292) of the high school students lived in villages or small towns < 10,000 inhabitants. Among the medical students, 53.8% (n = 399) came from rural areas and 46.2% (n = 343) from urban areas.

### Knowledge of TC

**Figure 1:**
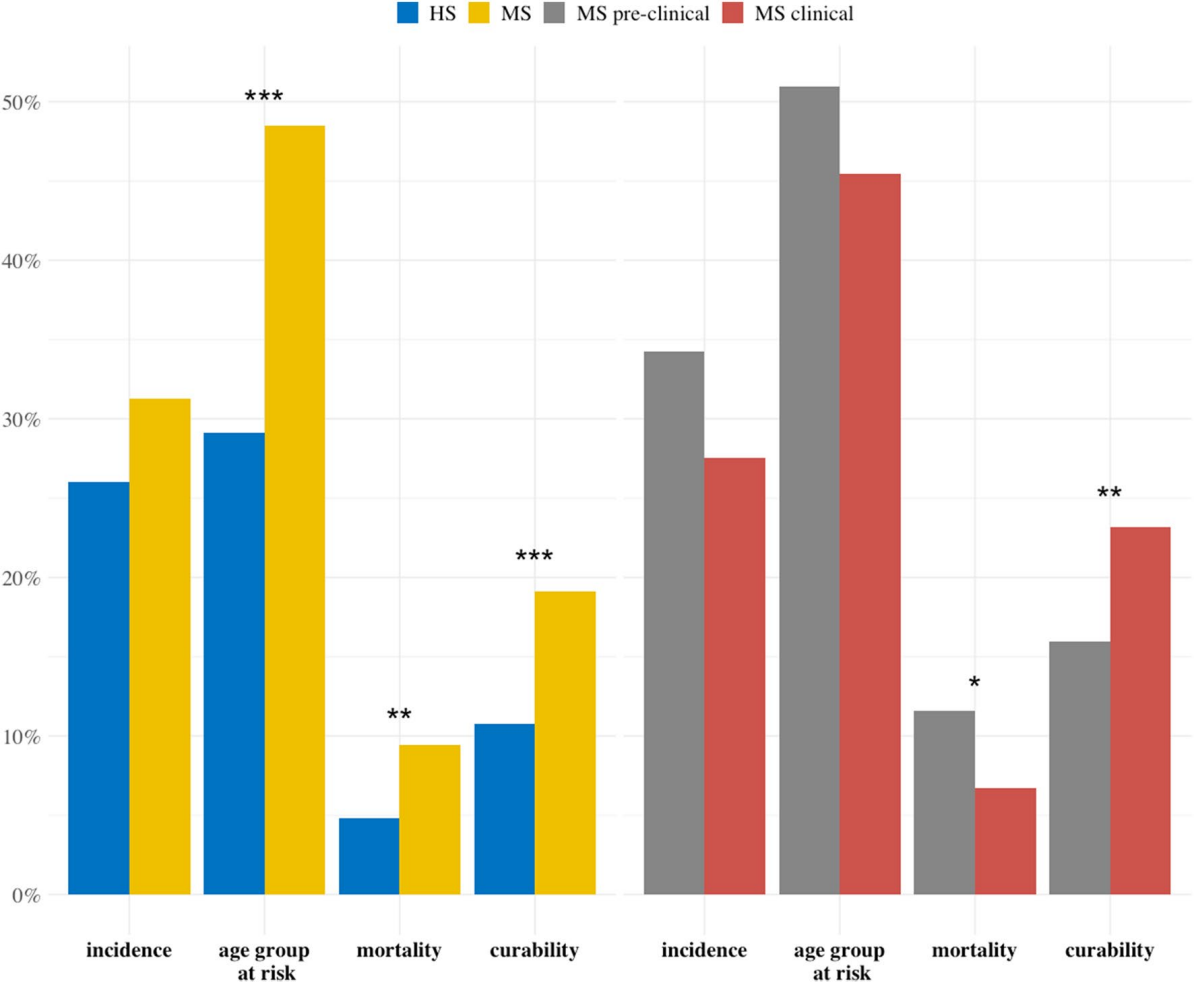
Young men's knowledge of the epidemiological statistics concerning testicular cancer; data are shown as a percentage of correct responses. * *p* ≤ 0.05; ** *p* ≤ 0.01; *** *p* ≤ 0.001; Current statistics: incidence in Europe: 5.6 per 100,000; age group at risk: 15–44 years; mortality in Europe: 0.4 per 100,000; curability: more than 90% if detected at an early stage^22^.

The term ‘testicular cancer’ was easy to comprehend for 71.9% and 98.1%-100% of the high school medical students, respectively. The knowledge of TC epidemiological facts was low in both the high school and medical students (Fig. 1); however, a significantly greater number of correct answers were provided by the medical than high school students. The age group at risk for TC was correctly recognized by only 29.1% of the high school students and 48.5% of the medical students (*p* ≤ 0.001). The proportion of proper awareness of the morbidity of TC was very low, i.e. only 4.8% of the high school respondents and 9.4% of the medical students (*p* ≤ 0.01) answered the question correctly.

The knowledge of the risk factors of TC is presented in Table 1. A low percentage (approx. 20–38%) of the participants correctly identified a majority of well-known TC risk factors. The family history was well recognized by both the high school and medical students (65.7% vs. 89.2%; *p* < 0.001) among factors that may increase the risk of disease. A high proportion of the participants incorrectly defined several TC risk factors, i.e. poor hygiene of intimate areas (15.0%-37.9% of students) or the use of heated seats (22.4–26.1% of students). Cryptorchidism was very poorly recognized as a TC predisposing factor; however, it was significantly more frequently indicated by the medical than high school students (37.9% vs. 20.9%; *p* < 0.001).

**Table 1:** Young men's knowledge of the TC risk factors; data are shown as percentages of selected answers that agreed with the statements regarding the increased risk of cancer development.

Risk factors for testicular cancer	HS (%)	MS (%)	*p*	Pre-clinical MS (%)	Clinical MS (%)	*p*
**Proven risk factors**						
Cryptorchidism	20.9	37.9	< 0.001	38.2	37.5	0.913
Family history of TC	65.7	89.2	< 0.001	91.5	86.3	0.029
HPV infection	32.5	29.6	0.378	28.7	30.8	0.599
**Unproven risk factors**						
High sexual activity	20.6	11.5	< 0.001	11.6	11.3	0.986
Androgyne intake	21.8	9.0	< 0.001	6.3	12.5	0.005
Lack of intimate places’ hygiene	37.9	15.6	< 0.001	15.0	16.5	0.651
Use of heated seats	22.4	26.1	0.214	35.0	14.9	< 0.001

HS, high school students; MS, medical students.

*p* value: two proportion Z-test - HS versus MS and pre-clinical MS versus clinical MS.

The level of education was clearly associated with the awareness of the methods for early detection of testicular cancer (Table 2). The awareness of TSE as a method for early detection of the pathology was very low among the high school students (only 25.1% of participants). A high number of both the high school and medical students indicated a visit to the doctor among the methods for early detection of TC (70.1% vs. 67%, respectively).

**Table 2:** Young men's knowledge of the testicular cancer diagnostic methods; data are shown as percentages of selected answers that agreed with statements regarding the acknowledged methods for TC early-stage detection.

Method for early detection of testicular cancer	HS (%)	MS (%)	*p*	Pre-clinical MS (%)	Clinical MS (%)	*p*
Medical visit and examination performed by the physician	70.1	67.0	0.337	62.6	73.8	0.001
Self-examination	25.1	87.6	< 0.001	89.4	85.4	0.126
Chest X-ray	8.1	8.1	1	9.7	6.1	0.103
Urine test	36.1	16.4	< 0.001	16.7	16.2	0.932

HS, high school students; MS, medical students.

*p* value: two proportion Z-test - HS versus MS and pre-clinical MS versus clinical MS.

The knowledge of the signs and symptoms of TC was significantly higher among the medical students, compared to the high school students (Table 3). For example, the proportion of proper answers about the presence of a palpable mass in the testicle as an important symptom for TC was by approx. 20% higher in the group of the medical students than in the high school group. There was relatively low recognition of induration of the testicle (48.1%) and scrotum enlargement (37.9%) by high school students. The medical students were more aware of pathologies that can mimic testicular cancer, e.g. spermatocele, hydrocele, or varicocele (Table 4).

**Table 3:** Young men's knowledge of testicular cancer signs and symptoms; data are shown as percentages of selected answers that agreed with statements regarding the common warning signs of TC.

Symptoms of testicular cancer	HS (%)	MS (%)	*p*	Pre-clinical MS (%)	Clinical MS (%)	*p*
Palpable mass in the scrotum	50.1	84.9	< 0.001	87.0	82.3	0.099
Induration, feeling of heaviness in the scrotum	48.1	82.3	< 0.001	80.9	84.1	0.294
Scrotum enlargement	37.9	66.7	< 0.001	67.9	65.2	0.499
Testicular pain, discomfort	46.3	16.2	< 0.001	19.8	11.6	0.003

HS, high school students; MS, medical students.

*p* value: two proportion Z-test - HS versus MS and pre-clinical MS versus clinical MS.

**Table 4:** Young men's knowledge of pathologies mimicking testicular cancer; data are shown as percentages of selected answers that agreed with statements regarding pathologies that can mimic TC.

Other pathologies mimicking testicular cancer	HS (%)	MS (%)	*p*	Pre-clinical MS (%)	Clinical MS (%)	*p*
Orchitis	57.3	42.5	< 0.001	35.7	50.9	< 0.001
Spermatocele	38.5	67.3	< 0.001	70.5	63.1	0.039
Varicocele	11.3	40.7	< 0.001	42.3	38.7	0.367
Hydrocele	35.8	49.7	< 0.001	46.4	54.0	0.048
Groin hernia	19.4	21.6	0.468	23.9	18.6	0.097
Twist of spermatic cord	16.4	23.2	0.015	20.3	26.8	0.045

HS, high school students; MS, medical students.

*p* value: two proportion Z-test - HS versus MS and pre-clinical MS versus clinical MS.

### Students' knowledge and attitudes towards testicular self-examination (TSE)

**Figure 2:**
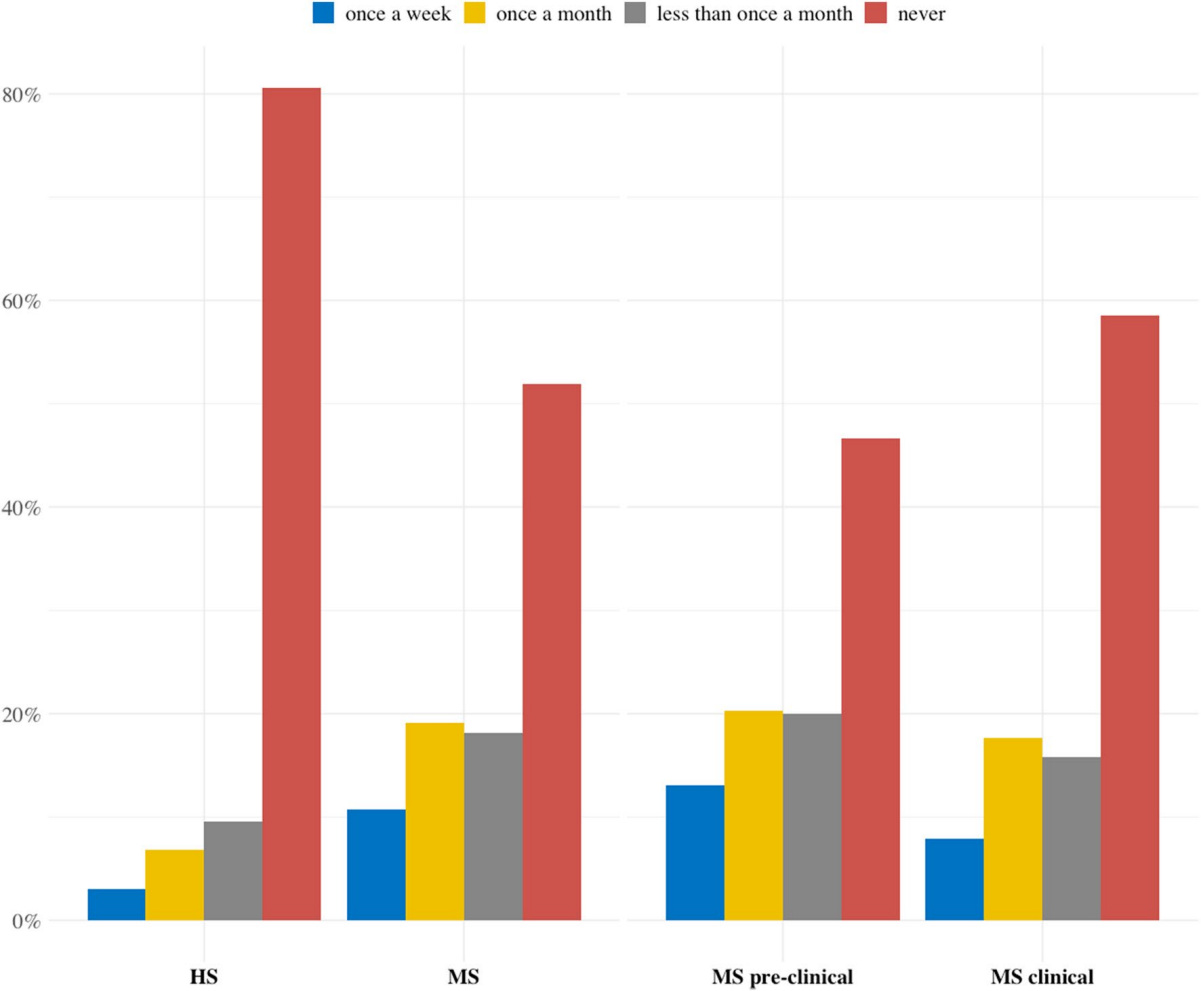
Young men's attitude towards testicular cancer self-examination; data are shown as a frequency of TSE.

The awareness of testicular self-examination (TSE) as an important method for early diagnosis and cancer detection was very weak. A majority of the respondents (> 80% of the high school respondents and > 50% of the medical school students) have never performed TSE (Fig. 2). Among the students who declared the TSE practice, the frequency of TSE was significantly higher in the group of medical students in comparison to group of high school students. However, the declared frequency of TSE was low; only 30% of the medical students and 9.9% of the high school students have practiced TSE once a month.

The most common reason for not performing TSE among both the high school and medical students was the lack of skills and knowledge how to perform the examination (Table 5). Greater numbers of the high school than medical students were afraid to detect cancer while performing self-examination (20.3% vs. 11.6%, respectively). In general, the students had a positive attitude towards TSE, and only 5.5–6.9% declared that ethical issues were a barrier for regular self-check-ups. Only a small percentage of the respondents (5.1% of the high school participants and 8.6% of the medicine students) have had the opportunity to attend professional training on TSE run by qualified teachers. A majority of the responders were willing to learn more about testicular cancer and testicular self-examination.

**Table 5:** Young men's altitude towards testicular cancer self-examination; data are shown as percentages of selected answers that agreed with statements regarding the reasons for not performing of TSE.

Reasons for not performing testicular self-examination	HS (%)	MS (%)	*p*	Pre-clinical MS (%)	Clinical MS (%)	*p*
I do not know how to perform TSE	63.0	45.6	< 0.001	47.8	42.7	0.186
TSE is irrelevant	19.7	28.6	0.003	18.1	41.8	< 0.001
I am afraid of detecting cancer	20.3	11.6	< 0.001	11.1	12.2	0.732
I am ashamed of performing TSE	8.4	7.5	0.736	9.7	4.9	0.021
Performing TSE is unethical	6.9	5.5	0.470	4.6	6.7	0.275

HS, high school students; MS, medical students.

*p* value: two proportion Z-test - HS versus MS and pre-clinical MS versus clinical MS.

### Knowledge and attitudes expressed by pre-clinical versus clinical medical students

The evaluation of the medical students' knowledge showed differences between the pre-clinical and clinical individuals (Tables 1, 2, 3, 4). For example, higher competence in answers concerning the morbidity of TC was demonstrated by the pre-clinical students, compared to their clinical counterparts (11.6% vs. 6.7%, respectively; *p* ≤ 0.05); on the contrary, better knowledge of TC curability was shown by the clinical than pre-clinical group (23.2% vs. 15.9%, respectively; *p* ≤ 0.01).

A significantly higher proportion of the pre-clinical students than the clinical students recognized family history among the TC risk factors. Among the unproven risk factors, androgen was indicated as a TC risk factor significantly more frequently by the clinical students compared to the pre-clinical group (12.5% vs. 6.3%; *p* = 0.005). In turn, the use of heated seats was indicated as an important factor in determination of the risk of TC by a higher proportion of the pre-clinical students than those in clinical training (*p* < 0.001).

The answers to the question of the TC signs and symptoms were similar in both groups. However, a testicular pain and discomfort as a testicular cancer symptom was indicated significantly more often by the pre-clinical students compared to the clinical group (19.8% vs. 11.6%; *p* = 0.003).

## Discussion

The study of young males’ knowledge and awareness of TC is highly relevant, as the rate of the TC incidence exhibits a constant rising tendency worldwide, including Poland^23,24^. Increasing awareness of risk factors, TC signs and symptoms, and methods for early diagnosis in young males is a significant part of the strategy for a decrease in TC morbidity and mortality^1^.

To our knowledge, the present study is the first report assessing the knowledge and awareness of TC as well as the attitude and practice of TSE among young males in Poland. Here we used a self-designed questionnaire to compare the knowledge and attitudes of high school and university medical students in order to forward the information about possible knowledge gaps to the appropriate authority and health policy-makers. Moreover, we analyzed the awareness of medical students at remarkably different levels of medical education to identify inadequacies in their knowledge of TC for development of better education curricula.

In our study, the percentage of participants that had heard about testicular cancer was quite satisfying (70–100%); nevertheless, the knowledge of the epidemiology and the awareness of risk factors responsible for the TC were low; only 30–50% of the respondents recognized these issues properly. The analysis of the literature showed that young males' knowledge of TC varied. Sugisaki et al. presented data suggesting that even approx. 50% of high school male respondents have never heard about TC^25^. Studies conducted in different world regions have highlighted a relatively low level of young males' awareness of TC, epidemiological facts and risk factors, as well as signs and symptoms^4,15,26^. Awareness of epidemiology and perception of risks are very important, as they are usually associated with motivation to reduce a possible danger and reinforce changes in behavior. They can also stimulate actions towards direct prevention and control, i.e. self-examination. Equally important, the consciousness that TC is a curable cancer should encourage young males to attend primary care service at an earlier stage, potentially improving the odds of survival and the financial aspect of health care related to more cost-effective treatment.

Our study showed inadequate knowledge of the well-known risk factors for testicular cancer. Regrettably, a high percentage of the participants defined the TC risk factors incorrectly, i.e. indicating poor hygiene of intimate areas or use of heated seats among possible risks for TC. In Northern Ireland, males aged 18–45 years, also falsely recognized TC risk factors, i.e. specifying body weight (44% of respondents) or alcohol drinking (21% of respondents)^20^. The awareness of cryptorchidism, which is a well-known risk factor for testicular cancer, was unsatisfying among our study population. Similarly, Vadaparampil et al. reported very low awareness of the link between cryptorchidism and TC^27^. This testicular abnormality is one of the most strongly established TC risk factors^28^. It has been proven that a personal history of cryptorchidism can increase the risk of TC up to 8-fold^29^. Therefore, it is particularly important to improve and raise students' awareness of risks associated with an undescended testicle.

A low level of awareness of young age as a risk factor for TC was revealed. Various reports have indicated that young male students are unaware of being in the age-risk group for TC, which is the most common neoplasm in 15–35 year age group^20,30,31^. Presumably, young men are not very conscious of cancer and their perception of malignant pathologies is associated only with older people^32^. Likewise, men can feel awkward about the male genital system and/or reproductive health and for that reason they can delay or even justify not visiting a doctor. It is also well known that patients who are unconscious of TC disorders may ignore testicular pain, lumpiness, and swelling^33,34^. These pathologies may be generally considered trivial symptoms linked to exercising activities or sport injuries. Regrettably, an emotional attitude, lack of awareness of TC symptoms, or underestimation of symptoms can have a harmful effect on men’s determination to solve the problem and seek medical help^34^.

It is optimistic that the family history of TC was indicated as a predisposing factor for developing TC. Men with a family history of TC have an approx. 4–9 times greater risk of TC^27^. The awareness and cancer-related concern deriving from family history may function as a facilitator of preventive behavior and should mobilize men to practice regular self-examination. Regular TSE is recommended by National Cancer Societies worldwide for men with a known family history to increase the chance of early detection of TC^35,36^. It is widely evidenced and agreed that men with a personal or family history of testicular syndromes and those with an inherent health-seeking behavior seemed to be better informed about testicular problems and had better conviction to request professional help for testicular symptoms^34,37^.

Our participants displayed no clear understanding of the signs and symptoms for TC. There was a high recognition of i.e. palpable mass in the testicle, induration of the testicle, or scrotum enlargement. Regrettably, the other crucial TC symptoms were recognized less often. Surveys conducted by various researchers also show that the percentage of correct answers concerning the early symptoms of TC fluctuates and seems to be unsatisfactory. Braga et al. found that only about 42% of Portuguese male respondents indicated to the most common TC signs and symptoms correctly^30^. Roy et al. documented that less than 30% of Irish men properly recognized the first symptoms of the disease^20^. Even lower awareness of the first TC signs was reported among Nigerian respondents, i.e. a lump in the testicle was identified by only approx. 10% of males. Moreover, they were not able to identify any other potential signs and symptoms of testicular malignancy^18^. Our survey and the study mentioned above emphasized that the low awareness of TC signs and symptoms derive from gaps in cancer education.

Self-examination is a beneficial tool to detect testicular cancer at an early stage, which gives a chance for a relatively high cure rate. It is estimated that approx. 90% of both benign and malignant testicular pathologies are detected by males performing regularly self-examination^38^. Therefore, it is important for young men to be aware of the possibility of early TC detection and to know how to perform a checkup. Fortunately, public health initiatives promote TSE and clearly indicate that close attention should be concentrated on the need to raise men’s awareness of TC in order to reduce tumor size at presentation^39^. Meanwhile, an extensive survey of Saab et al. established that although men had heard of TC, they had no knowledge of different traits of this malignancy^37^. Low awareness of TC and non-malignant disorders (i.e. epididymitis, testicular torsion, orchitis) may have an adverse effect on the search for professional medical help. If left untreated, all these syndromes can cause numerous complications or can even be life-threatening conditions. Therefore, efforts should be made to raise the awareness of the potential significant advantages of TSE, early TC symptoms, and other testicular disorders^40,41^.

The high level of awareness of the TSE practice was characteristic only for our medical students. In general, the literature reports indicate that the state of knowledge and awareness related to testicular self-examination in the young male population is insufficient. For example, TSE is regularly performed by 2% of young men in Denmark, 1.9% of young males in Turkey, 8.5% of males in Iran, 12.3% of male students in France^14,42–44^. The testicular self-examination was found to be most popular among British, Irish, and Hungarian students; however, the rate is still very low, with only approx. 25% of individuals performing TSE^16,45^. Therefore, it is crucial to promote and develop the awareness about TSE as an early cancer detection method in young adult men.

The main reason for not performing self-examination was the lack of knowledge and practical skills. However, 85% of our students are willing to learn more about the methods for testicular cancer prophylaxis^44,46^. Similarly, our students never tried to perform TSE due to the lack of knowledge how it should be done. The importance of professional training on the TSE practice was revealed in the United Kingdom and the United States, where the TSE performance ratio among male college students increased from 58.4 to 69.3% and from 9 to 36%, respectively, after practical professional training^42,47^. It should be emphasized that the most effective education programs on testicular self-examination should match the age and the educational level of the recipients. It is crucially important that programs regarding male genital diseases should be prepared and conducted by well-trained teachers, who should employ interactive tools instead of standard theoretical training^21^. Moreover, the teachers should be empathic and avoid cheeky humor or jokes regarding genitals^44^. Currently, the education can be delivered through a number of tools, i.e. television shows, campaigns, the internet or, to a lesser extent, print media (newspapers, magazines, billboards, posters, or direct mail). In particular, the use of social media (i.e. Facebook, Instagram, YouTube) in education can be extended. However, gender preference should be taken into account^41^. There are recommendations to use multimodal learning strategies (i.e. auditory*,* visual, kinesthetic) or consider simulations rather than to use diagrams or printed graphs while developing training programs and materials for men^48^. These suggestions are resonated in literature data on the TSE promotion, i.e. Thornton established that campaigns engaging written materials have been ineffective^49^. Mobile phone applications should also be used to improve the awareness of testicular disorders among young men. However, school and university campaigns are still adequate mechanisms to promote awareness of TC, self-examination, and screening^37,41^.

In general, our study reflects the deficiencies in health education among high school and medical students. As expected, the high school students had poorer knowledge and awareness of the epidemiological facts, risk factors, signs and signals of TC, and TSE than the medical students. Many studies demonstrated that the education level was a major determinant of cancer knowledge, and increased awareness was associated with a higher level of education^20,30,50^. The awareness of cancer can be also associated with several other factors, i.e. income, ethnicity, or social position^51^.

It is worth mentioning that our pre-clinical students were more conscious of the TC incidence rate, age risk group, and morbidity than the clinical students. At the Medical University in Lublin, as in other medical universities, the pre-clinical modules consist of basic sciences (i.e. anatomy, histology, physiology, epidemiology and hygiene, pathomorphology, and pathophysiology). They are focused on teaching the pathology of diseases, epidemiology, disease prevention, population health, and health promotion^52^. In the clinical stage, the students are exposed to patients in different hospital wards, gain history-taking competence, and have an opportunity to acquire clinical skills. Various experiments have revealed that medical students forget approx. 25–35% of basic science knowledge after 1 year and more than 50% after the following year^53,54^. Cancer is a major public health problem; therefore, medical students are expected to remember details of the disease for a longer time. Therefore, there are several possible approaches to consider in order to increase the medical students' knowledge: (1) the module programs should be rearranged, (2) more emphasis should be placed on cancer epidemiology, risk factors, and prevention, (3) teaching should be remodeled and more attention should be paid to practical skills, (4) teachers have to improve their teaching methods by implementation of i.e. simulation techniques, clinical cases, or problem-based teaching. However, equally important is that students have to be mobilized to advance their learning methods.

It has to be emphasized that our study has several limitations. First, the data were collected in only one region of Poland. Secondly, the medical students attended only one medical school. Third, the study was performed using researcher-designed questionnaire. However, according to our knowledge there is lack of standardized questionnaire assessing cancer knowledge. Hence, the experience of the participants may not fully reflect what may be obtainable in the general population of young man. However, the survey shows preliminary data that can be useful in the design of further testicular cancer programs and services.

## Conclusions

Our study has revealed that the level of knowledge of TC among students, including medical students, is not satisfying. Therefore, it is still a great task for health education with respect to TC and efforts should be made to construct programs aimed at improving the knowledge and awareness of TC. Raising the awareness of TC among most at-risk young men may reduce the fear of development of TC cancer and change the attitude towards the first signs and symptoms as well as self-examination, which may contribute to early diagnosis. Schools are a valuable asset in health education, and cancer prevention modules should be included in school curricula as well as employment of modern technology and social media.

## Methods

### Study design and participants

In total, 1077 male students from high schools (n = 335; HS) and medical university students (n = 742; MS) participated in the survey. Individuals who were (1) male, (2) residents in the Republic of Poland, and (3) aged 14–50 years, i.e. men who are at the highest risk of developing TC, were eligible for the inclusion criteria. The medical students were divided into two subgroups: (1) pre-clinical medical students (MS pre-clinical; first- to third-year students; n = 414) and (2) clinical medical students (MS clinical; fourth- to six-year students; n = 328). The participants came from SE Poland. The high school students attended public schools, and all medical students were members of the Faculty of Medicine of the Medical University of Lublin.

The study involved the completion of an original questionnaire written in Polish. The first version of the questionnaire included 30 items designed as a multiple-choice question type and ‘yes’ and ‘no’-type questions. Prior to designing the questionnaire, an exhaustive literature review had been conducted. Next, a content validity survey was generated to all questionnaire items (each item was assessed using a three-point scale: not necessary, useful but not essential, and essential). The questionnaire with the survey was sent to the Content Evaluation Panel (panel of experts in the field of research: two urologists, surgeon, psychologist, public health specialist, and biostatistician). The content validity ratio (CVR) was then calculated for each item by employing Lawshe’s method^55^. CVR of 0.78 or higher was considered evidence of good content validity^56^. Four items did not reach this threshold and were deleted from the final instrument.

The finally approved paper-and-pen questionnaire consisted of 26 questions divided into 2 main parts. The first part (14 questions) aimed to assess the participants’ knowledge and awareness of testicular cancer (TC), i.e. the epidemiology, cancer risk factors, signs and symptoms, and methods for early detection. Data concerning epidemiological statistics were drawn from the Cancer registry system in Poland and the study conducted by Park et al.^22,23^. The second part (12 questions) was designed to obtain students’ opinion about testicular self-examination (TSE), their attitudes towards performing TSE, and their willingness to get more knowledge of TC and TSE practice.

The study was conducted according to general ethical standards; the questionnaire was filled anonymously and voluntarily. The study was approved by the Ethical Committee of the Medical University of Lublin and was conducted in accordance with the Declaration of Helsinki. The informed consent was obtained from all participants or their legal guardian if participants age was below 18. Prior to the questionnaire distribution, the participants had been informed about the objectives of the study; additionally, the basic information about the aim of the study was provided at the top of the questionnaire. Then, questionnaires were distributed to the students who had agreed to participate. In total, 1158 questionnaires were therefore distributed and 1077 completed questionnaires were returned, giving an overall response rate of 93%. The time given to fill out the questionnaire was 20 min.

### Statistical analysis

The participants' characteristics underwent a descriptive analysis. Continuous variables were presented as means ± standard deviations (SD), and categorical variables were shown as the numbers and percentages of individuals. A two proportion Z-test was used to compare the answers of the groups of students concerning the knowledge of epidemiological facts, early diagnostic methods, risk factors, signs and symptoms, and reasons for not performing TSE. The frequency of TSE was compared between the groups using a Chi-square test for homogeneity. Differences with a p-value less than 0.05 were considered significant. The data were explored and analyzed using the RStudio ver. 1.1.463 software (Boston, MA, USA).
